# Repair of Posterior Vaginal Wall Defect Using Biologic Graft for Stage III-POP-Q Pelvic Organ Prolapse in a Patient with History of a J-Pouch

**DOI:** 10.1155/2020/8892014

**Published:** 2020-07-27

**Authors:** Ali Azadi, Stephanie Casey, Paravasthu Ramanujam, Darya Tehranchi, Donald Ostergard

**Affiliations:** ^1^Star Urogynecology, Peoria, AZ, USA; ^2^University of Arizona College of Medicine, Phoenix, AZ, USA; ^3^Saint Barnabas Medical Center, Livingston, NJ, USA; ^4^West Valley Colon and Rectal Surgery Center, Suncity, AZ, USA; ^5^Arizona College of Osteopathic Medicine, Glendale, AZ, USA; ^6^UCLA School of Medicine, Los Angeles, CA, USA

## Abstract

Surgical correction is considered in women with symptomatic pelvic organ prolapse (POP). There is an expected increase in the prevalence of surgical correction due to an aging population within the United States. Individuals with previous colorectal surgery present a unique challenge considering the changes in pelvic anatomy. This case discusses the challenges of posterior colporrhaphy in a patient with previous, remote J-pouch surgery. In traditional posterior colporrhaphy, randomized controlled trials have not shown any benefit of graft augmentation (Maher, 2016). However, the utilization of a biologic graft to improve anatomical correction is discussed in this unique case. Short term anatomical success was obtained without immediate complications in the postoperative period. In a patient with a history of ulcerative colitis with colorectal resection and a J-pouch, surgery can be challenging due to alterations of pelvic anatomy. Modification of the standard surgical approach may be required to achieve success.

## 1. Introduction

Pelvic organ prolapse (POP) prevalence increases with age with currently 13% of women undergoing surgical correction within their lifetime [1]. There is an anticipated increase in the incidence of women pursuing surgical correction due to an aging population within the United States [2]. Pelvic organ prolapse is the descent of one or more aspects of the vagina and uterus: the anterior vaginal wall, the posterior vaginal wall, the uterus (cervix), or the apex of the vagina (vaginal vault or cuff scar after hysterectomy) [3]. Changes in pelvic support allows nearby organs to herniate into the vaginal space, which is commonly referred to as cystocele, rectocele, or enterocele.

In one recent study, 53% of women presenting with POP had a posterior vaginal wall component [4]. The presenting symptoms and severity tend to be associated with the organ(s) involved in the prolapse with worsening severity of symptoms with increasing grade/stage of POP. Options for management of POP include observation, lifestyle modification to decrease modifiable risk factors for worsening prolapse, management of the predominant symptoms, physical therapy for the pelvic floor, and pessary placement; failure of the above management leads to the pursuit of surgical correction. Surgical management can be done via the vaginal or abdominal approach. Traditionally, posterior colporrhaphy with native tissue has been done for symptomatic posterior vaginal wall prolapse. It has the advantage of a vaginal retroperitoneal approach with avoidance of abdominal incisions.

**Figure 1:**
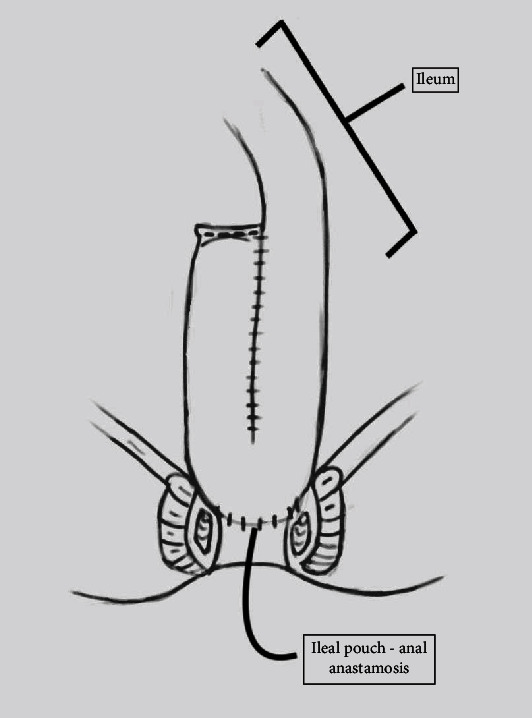
Representation of ileal pouch-anal anastomosis (J-pouch formation) utilizing small bowel to create a pouch after the colon and rectum have been removed. (Image courtesy of Hannah Mitchell).

A variety of surgical approaches have been described for correction of a posterior vaginal wall defect, however, randomized controlled trials have not shown improvement in outcomes with the insertion of a graft in the posterior compartment [5]. Here, we introduce a case of a patient with stage III posterior wall prolapse with symptoms of fecal incontinence, obstructive defecation, pelvic pressure, and vaginal bulge with a history of a J-pouch for restorative proctocolectomy due to ulcerative colitis. A J-pouch was created by looping two segments of the small intestine and attaching them to the rectal cuff (Figure 1). Potential complications from J-pouch creation include pouch fistula, anastomotic stricture, pouchitis, prolapse of the pouch, irritable pouch syndrome, and pouch inertia [6]. The absence of the rectum and the inability to create the rectovaginal dissection normally utilized for posterior colporrhaphy created a situation with a challenging dissection for this patient's surgical repair.

## 2. Case

**Figure 2:**
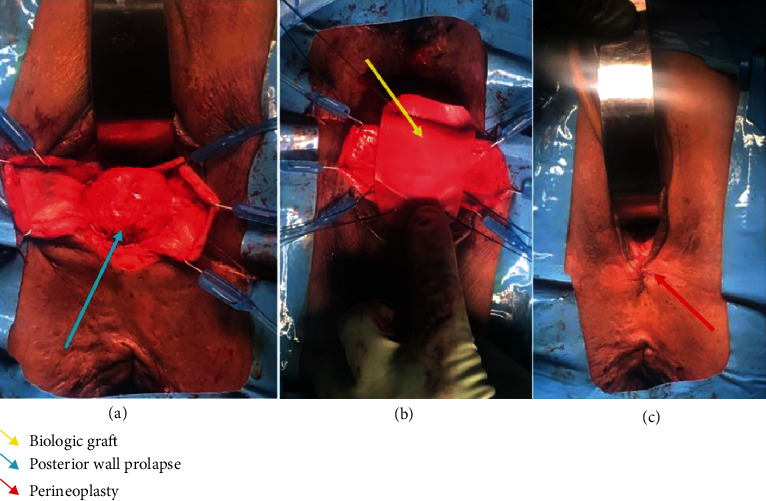
(a) Intraoperative image detailing posterior vaginal wall prolapse after dissection and retraction of the anterior vaginal wall. The retractor is pictured anteriorly. (b) Insertion of bovine biologic graft with iliococcygeal suspension sutures for repair of posterior and apical vaginal wall prolapse. (c) Postoperative anatomic resolution of posterior and apical vaginal wall defects with concomitant perineoplasty.

A 70-year-old Caucasian woman and Body Mass Index (BMI) of 24 kg/m^2^ with symptomatic stage III POP presented to our office for evaluation for surgical correction due to failed conservative measures. She previously underwent colectomy 24 years ago, along with subsequent partial small bowel removal with a resultant J-pouch, and hysterectomy. The patient described fecal incontinence, obstructive defecation, pelvic pressure, and a vaginal bulge. POP-Q evaluation indicated the International Continence Society (ICS) stage III Bp prolapse with the involvement of apical prolapse (Point C at 0) (Figure 2(a)). After reviewing the options, she decided to proceed with a vaginal repair for the posterior wall prolapse.

Due to extensive adhesions and scarring, the dissection of the small bowel from the vaginal epithelium and muscularis proved to be difficult as previously anticipated. The dissection of the posterior vaginal wall extended to the mid vagina, but we were unable to extend the dissection all the way to the apex due to dense adhesions at the pouch. The sacrospinous ligaments were unable to be reached due to a concern for bowel damage with further dissection. Instead, the iliococcygeal muscles were reached and utilized for bilateral vault suspension. Delayed absorbable sutures (Polydiaxonone) were used for suspension and then anchored to a segment of tailored biological graft at each corner. A segment of tailored biologic bovine graft was used with appropriate tension on the suspension sutures to reduce the posterior bulge (Figures 2(b) and 2(c)). After suspension sutures were tied, the vaginal bulge was reduced and excellent support obtained. The edges of the vaginal epithelium were trimmed and the incision was closed using running absorbable sutures. Perineoplasty was performed at the end of the surgical procedure. Cystoscopy and endoscopy were performed intraoperatively to confirm patency of ureters and integrity of the bladder and bowel.

## 3. Results

The patient had a noncomplicated postoperative course. Anatomical evaluation at 6 weeks and 6 months postoperatively showed resolution of the posterior vaginal wall prolapse and its symptoms.

## 4. Discussion

Due to our patient's history of J-pouch status post colectomy and removal of the rectum in addition to surgical management of a previous small bowel obstruction, the surgical correction of her vaginal prolapse was anticipated to be challenging. Despite extensive scarring between the J-pouch and posterior vaginal wall as seen in this case, the patient developed a stage III posterior vaginal wall prolapse. Our patient opted for surgical management due to the severity of symptoms and lack of improvement with conservative management. The surgical management in these patients is challenging due to the alteration of normal anatomy and extensive scarring which makes it difficult to identify important anatomical landmarks and fascial planes intraoperatively. To our best knowledge, the available literature is limited to the prolapse of J-Pouch without any description of the repair of posterior vaginal wall defect in patients with J-pouch. The use of synthetic mesh or biologic graft augmentation has shown the benefit of increasing anatomical success with a reduced rate of recurrence with use in anterior vaginal wall repair [5]. Clinical trials have shown no evidence of benefit to utilizing a graft over traditional methods for posterior wall repair [7, 8]. Further research is needed to determine the effectiveness of graft versus native tissue repair in patients with previous colorectal surgeries with resultant alterations of the anatomy in the posterior vaginal wall. Research further quantifying the effectiveness and complications of surgical POP repair has been complicated further by graft products previously studied no longer being available. In this case, the use of a biologic graft and iliococcygeal suspension were a beneficial alternative to traditional surgical treatment with native tissue due to our patient's alterations in anatomy and the surgical limitations in dissection secondary to her previous surgical history. Even though posterior repair with graft has not been shown to be superior to native tissue repair, our case suggests that the use of a graft can be considered for correction of posterior wall defects with altered anatomy, such as in patients with a history of J-pouch surgery. Our case indicates that this approach adequately addressed apical and posterior vaginal prolapse in a patient with a history of a J-pouch achieving short term success without any adverse outcomes.

## Data Availability

Any case information may be obtained from the clinical records of Star Urogynecology.
